# Evaluating treatment effectiveness under model misspecification: A comparison of targeted maximum likelihood estimation with bias-corrected matching

**DOI:** 10.1177/0962280214521341

**Published:** 2014-02-12

**Authors:** Noémi Kreif, Susan Gruber, Rosalba Radice, Richard Grieve, Jasjeet S Sekhon

**Affiliations:** 1 Department of Health Services Research and Policy, London School of Hygiene and Tropical Medicine, London, UK; 2 Department of Epidemiology, Harvard School of Public Health, Boston, MA, USA; 3 Birkbeck, University of London, London, UK; 4 Travers Department of Political Science, UC Berkeley, Berkeley, CA, USA

**Keywords:** targeted maximum likelihood estimation, bias-corrected matching, treatment effectiveness, machine learning, double robustness, model misspecification

## Abstract

Statistical approaches for estimating treatment effectiveness commonly model the endpoint, or the propensity score, using parametric regressions such as generalised linear models. Misspecification of these models can lead to biased parameter estimates. We compare two approaches that combine the propensity score and the endpoint regression, and can make weaker modelling assumptions, by using machine learning approaches to estimate the regression function and the propensity score. Targeted maximum likelihood estimation is a double-robust method designed to reduce bias in the estimate of the parameter of interest. Bias-corrected matching reduces bias due to covariate imbalance between matched pairs by using regression predictions. We illustrate the methods in an evaluation of different types of hip prosthesis on the health-related quality of life of patients with osteoarthritis. We undertake a simulation study, grounded in the case study, to compare the relative bias, efficiency and confidence interval coverage of the methods. We consider data generating processes with non-linear functional form relationships, normal and non-normal endpoints. We find that across the circumstances considered, bias-corrected matching generally reported less bias, but higher variance than targeted maximum likelihood estimation. When either targeted maximum likelihood estimation or bias-corrected matching incorporated machine learning, bias was much reduced, compared to using misspecified parametric models.

## 1 Introduction

Health policy makers require unbiased, precise estimates of the effectiveness and cost-effectiveness of health interventions.^1–3^ When observational studies are used to estimate average treatment effects (ATEs), it is vital to address potential bias due to confounding. Most studies use methods that assume there is no unmeasured confounding.^4^ Under this assumption unbiased estimates can be obtained after controlling for observed characteristics, for example with parametric regression, propensity score (PS) or double-robust (DR) methods, provided either the PS or the endpoint regression model is correctly specified.

In studies that rely on regression methods alone, the estimated ATEs can be highly sensitive to the choice of model specification.^5^ When evaluating health care interventions, correctly specifying a regression model can be challenging. For example, health-related quality of life (HRQoL) data are often semi-continuous, with non-linear covariate–endpoint relationships.^6^ Instead, PS approaches may be preferred such as matching or inverse probability of treatment weighting (IPTW), but these rely on the correct specification of the PS. Most medical applications use a PS estimated with logistic regression models that only include main effects, which raises the concern of model misspecification.^7,8^

DR methods^9,10^ are consistent if either the endpoint regression or the PS is correctly specified. However, in practice both the regression function and the PS may be misspecified, and also, poor overlap can lead to the estimated PSs close to 0 and 1.^11^ Here, DR methods such as weighted regression may not protect from bias.^12,13^ A recently proposed DR method, targeted maximum likelihood estimation (TMLE),^14,15^ can be less biased and more efficient than conventional DR methods when there is poor overlap^16–18^ by respecting known bounds on the endpoint. Another approach which can exploit information from the PS and the endpoint regression is bias-corrected matching (BCM).^19,20^ This method aims to reduce residual bias by adjusting the matching estimator with regression predictions of the endpoint. BCM is relatively robust under misspecification, for example, unless the functional form relationship between the covariates and the endpoint is highly non-linear; adjustment using a linear regression for the endpoint can reduce most of the residual bias from imbalance in the matched data.^20–23^ However, an outstanding concern with TMLE and BCM that use fixed, parametric models is that there may be residual bias due to functional form misspecification of both the PS and endpoint regressions.

In order to minimise bias due to functional form misspecification, both methods can exploit machine learning techniques. Unlike fixed, parametric models, where the functional form is chosen by the analyst, these methods use an algorithm to find the best fitting model. Machine learning estimation approaches for estimating the PS^8,24,25^ and the endpoint regression function^26^ have been shown to reduce bias due to model misspecification. However, few studies have investigated machine learning for DR approaches.^16,27^ No previous study has considered machine learning for BCM.

This paper aims to compare TMLE and BCM and to combine both methods with machine learning for estimating the PS and the endpoint regression function. The methods are contrasted for estimating the ATE of a binary treatment, with a focus on dual functional form misspecification of the PS and the endpoint regression. We also compare TMLE and BCM to other commonly applied DR,^12^ PS matching^28,29^ and regression^6^ approaches.

We illustrate the methods in a motivating case study and in a simulation study. The case study considers the relative effectiveness of alternative types of total hip replacement (THR) on post-operative HRQoL for patients with osteoarthritis. We exploit a large UK survey, which collects patient reported outcome measures (PROMs),^30,31^ before and after common elective surgical procedures. This case study exemplifies the challenge of correctly specifying the endpoint regression function. The simulation study was grounded in the case study and compared the relative performance of the methods for a range of data generating processes (DGPs). We compare the relative performance of the methods according to bias, root mean squared error (RMSE) and coverage rates of nominal 95% confidence intervals (CIs).

In the next section, we outline the statistical methods under comparison. Section 3 presents the motivating example. Section 4 reports the design and results of the simulation study. The last section discusses the findings and suggests areas for further research.

## 2 Statistical methods

The parameter of interest is the ATE of a binary treatment *A*, defined as

ψ=E[Y(1)-Y(0)]

where Y(1) is the potential outcome under treatment, i.e. the endpoint that would be observed under the treatment state, and Y(0) is the potential outcome under the control state. The vector of confounding factors, that is all factors that influence the potential outcomes and treatment assignment, is defined as *W*. Under the assumption of no unmeasured confounders,^32^ all elements of *W* are observed, and the mean of the conditional distribution of the potential outcomes corresponds with the mean of the conditional distribution of the observed endpoint *Y*

E[Y(1)|W]=E[Y|A=1,W]   and   E[Y(0)|W]=E[Y|A=0,W]

Under the additional assumptions of consistency and positivity, the ATE can be identified as

ψ=E{E[Y|A=1,W]-E[Y|A=0,W]|W}

where the (potentially heterogeneous) individual level treatment effects are marginalised over the distribution of *W* The consistency assumption states that an individual’s potential outcome under the observed treatment is exactly the observed endpoint.^33^ The positivity assumption requires that there are both treated and control individuals at each combination of the values of observed confounders in the population,^11^ formally, 0<g(A,W)<1, for any stratum defined by *W*, where g(A,W)=P(A|W) is the model for the treatment assignment. In finite samples, positivity violations often arise; in particular covariate strata there might be few or no individuals from either treatment group,^11^ and so the estimated $\overset{\wedge }{g}(A,W)$ can be close to 0 or 1. The econometric literature on matching methods refers to positivity violations as ‘poor overlap’,^34^ and we use this terminology throughout the paper.

### 2.1 Regression estimators

We consider a general regression estimator, the G-computation estimator,^35^ which uses estimates of the expected potential outcomes, conditional on observed characteristics, defined as Q(A,W)=E[Y|A,W]. The estimator for the ATE is given by

(1) $${\overset{\wedge }{\psi }}^{\mathrm{reg}}=\frac{1}{N}\sum_{i=1}^{N}\{\overset{\wedge }{Q}(1,W_{i})-\overset{\wedge }{Q}(0,W_{i})\}$$

where $\overset{\wedge }{Q}(1,W)$ and $\overset{\wedge }{Q}(0,W)$ are the estimated potential outcomes for each individual under treatment and control states, respectively, and *N* is the number of subjects in the sample.

$\overset{\wedge }{Q}(0,W)$ and $\overset{\wedge }{Q}(1,W)$ can be obtained by fitting a regression model that includes the observed covariates and a treatment variable, for example ordinary least squares (OLS) regression or a generalised linear model (GLM). A more flexible method is to fit separate models for the treatment and control samples.^36^ When there is poor overlap, regression estimators extrapolate, which can lead to large biases if the regression model is misspecified.^5,37^

#### 2.1.1 Machine learning estimation of the regression function

In general, machine learning covers a wide range of classification and prediction algorithms.^8,26^ Unlike approaches that assume a fixed statistical model, for example a GLM with a log link, machine learning aims to extract the relationship between the endpoint and covariates through a learning algorithm.^24^ Machine learning approaches for estimating the endpoint regression can reduce bias which results from model misspecification.^26^ Here we consider the ‘super learning’ algorithm,^38^ which uses cross-validation to select a weighted combination of estimates given by different prediction procedures.^39^ The range of prediction algorithms is pre-selected by the user, potentially including parametric and non-parametric regression models. Asymptotically, the super learner algorithm performs as well as the best possible combination of the candidate estimators.^40^

### 2.2 PS methods

The PS, defined as the conditional probability of treatment assignment given *W*, g(1|W)=Pr(A=1|W), can create balance between the distributions of observed confounders of the treatment and control samples.^41^ The PS matching estimator imputes the missing potential outcomes, Y(0) or Y(1), for each individual, using the observed endpoints of the closest *M* individuals (the matches), where closeness is measured by the estimated PS, $\overset{\wedge }{g}(A=1,W)$

$$\hat{Y}(0,W_{i})=\{Y_{i}\text{if}A_{i}=0\frac{1}{M}\sum_{j\varepsilon \zeta_{M}(i)}Y_{j}\text{if}A_{i}=1\hat{Y}(1,W_{i})=\{\frac{1}{M}\sum_{j\varepsilon \zeta_{M}(i)}Y_{j}\text{if}A_{i}=0Y_{i}\text{if}A_{i}=1$$

and where $\zeta_{M}(i)$ is the set of *M* individuals from the opposite treatment group, matched to unit *i*. The estimator for the ATE is the mean of the estimated individual-level treatment effects

$${\overset{\wedge }{\psi }}^{\mathrm{match}}=\frac{1}{N}\sum_{i=1}^{N}\{\overset{\wedge }{Y}(1,W_{i})-\overset{\wedge }{Y}(0,W_{i})\}$$

IPTW reweights the treated and control samples using inverse weights $\frac{A_{i}}{\overset{\wedge }{g}(1|W_{i})}$ for the treated and $\frac{1-A_{i}}{1-\overset{\wedge }{g}(1|W_{i})}$ for the control observations. The normalised IPTW estimator^12,42^ is defined as

$${\overset{\wedge }{\psi }}^{\mathrm{IPTW}}=\frac{\sum_{i=1}^{N}A_{i}\frac{Y_{i}}{\overset{\wedge }{g}(1|W_{i})}}{\sum_{i=1}^{N}\frac{A_{i}}{\overset{\wedge }{g}(1|W_{i})}}-\frac{\sum_{i=1}^{N}(1-A_{i})\frac{Y_{i}}{1-\overset{\wedge }{g}(1|W_{i})}}{\sum_{i=1}^{N}\frac{1-A_{i}}{1-\overset{\wedge }{g}(1|W_{i})}}$$

Matching estimators are consistent if $\overset{\wedge }{g}(\cdot )$ is correctly specified,^43^ but can have finite sample bias, and are less precise than a correctly specified regression estimator.^19,44^ With a correctly specified $\overset{\wedge }{g}(\cdot )$, IPTW can also provide consistent and efficient estimates.^45^ However, poor overlap can result in unstable inverse probability of treatment (IPT) weights, and biased or inefficient estimates of the ATEs, even when $\overset{\wedge }{g}(\cdot )$ is correctly specified.^12,21,24,46^ In these settings, recommended approaches include stabilising IPT weights,^47^ truncating IPT weights at fixed levels^48^ or percentiles of $\overset{\wedge }{g}(\cdot )$^47^ as well as estimating ATEs for a subsample with good overlap.^49^

#### 2.2.1 Machine learning estimation of the PS

Instead of estimating the PS with a fixed parametric model, flexible approaches have been proposed to help correctly specify g(.). These include the series regression estimator,^45^ and methods from the machine learning literature, including decision trees, neural networks, linear classifiers and boosting.^8,25,50^ This paper considers boosted classification and regression trees (CART), as it has been shown to reduce bias in the estimated ATE compared to a misspecified logistic regression, and other machine learning methods such as pruned CARTs.^24^ When performing boosted CARTs, regression trees are fit on random subsets of the data, and in each iteration, the data points that were incorrectly classified with the previous trees receive greater priority. According to general recommendations,^51^ the algorithm can be set to select the final PS model that maximises covariate balance.^24,50^

### 2.3 DR methods

DR methods^9,52^ combine models for Q(.) and g(.), with most estimators using $\overset{\wedge }{g}(.)$ to construct IPT weights.^53^ The distinctive property of DR estimators is that they are consistent if either (but not necessarily both) *g*(ċ) or *Q*(ċ) is correctly specified.^9^ If both components are correct, the DR estimator can be a semi-parametric efficient estimator.^10,15^ A commonly used DR method is the weighted least squares (WLS) regression,^12,13^ which weights the covariates in a linear regression, using IPT weights.

In realistic settings such as when there is poor overlap and dual misspecification, weighted regression can report biased and inefficient estimates of ATEs.^12,13,16,54^ An ongoing debate discusses the relative merits of different DR estimators in these circumstances.^10,16,55^ One recommendation is to use machine learning methods to estimate *g*(ċ).^27^ A further suggestion is that DR estimators should have a ‘boundedness property’: they should respect the known bounds of the endpoint – for example that an HRQoL endpoint ranges from the value for the worst imaginable health state (−0.59) to that for full health, 1^56^ – so that the estimated parameter will always fall into the parameter space.^10,57^ This property can reduce bias and increase precision when the PS is used as weights, where large weights can lead to estimated values of the endpoint falling outside of a plausible range.^18^ Below we discuss a DR estimator, TMLE, that can have this boundedness property^57^ and is therefore appealing when overlap is poor.^18,58^

#### 2.3.1 TMLE

While standard maximum likelihood estimation aims to find parameter values that maximise the likelihood function for the whole distribution of the data, TMLE focuses on the portion of the likelihood required to evaluate the parameter of interest.^15,59^ This is achieved by using information in the PS to update an initial outcome regression, as described in the next section. The TMLE solves the efficient influence curve estimating equation, where an influence curve describes the behaviour of an estimator under slight changes of the data distribution.^60^ This results in the property of double robustness, and if both *Q*(ċ) or *g*(ċ) are correct, in semi-parametric efficiency.^14^

Performing TMLE involves two stages,^61^ which, for estimating the ATE, are

1. Obtain an initial estimate of the conditional mean of *Y* given *A* and *W*, by using regression to predict the potential outcomes *Q*(1,*W*) and *Q*(0, *W*).
2. Fluctuate this initial estimate, ${\overset{\wedge }{Q}}^{0}(A,W)$, by exploiting information in the treatment assignment mechanism.

Here, the fluctuation corresponds to extending the parametric model for Q(A,W) with an additional covariate *h*, which is a function of the PS

$$h(A,W)=\frac{A}{g(A=1|W)}-\frac{1-A}{1-g(A=1|W)}$$

In the extended parametric model, $Q^{1}(A,W)=Q^{0}(A,W)+ɛh(A,W)$, ɛ is fitted by maximum likelihood. h(A,W) is defined so that the solution of the score equation of this model implicitly also solves the efficient influence curve estimating equation for the ATE parameter. In practice, this translates to regressing the observed endpoint on *h* and an initial estimate $\overset{\wedge }{Q^{0}}(A,W)$ as offset. This regression can be interpreted as explaining any residual variability after the initial estimate, using information from the treatment assignment mechanism.

To ensure the boundedness of the TMLE, for continuous endpoints it is recommended that known bounds of the endpoint are exploited by rescaling *Y* to between 0 and 1.^18,58^ The rescaled endpoint is defined as $Y^{*}=\frac{Y-a}{b-a}$, where *a* and *b* are known limits of *Y*. Using Y*, $Q^{*}(A,W)=\frac{Q(A,W)-a}{b-a}$ can be defined. The fluctuation can then be performed on the logistic scale

$$\mathrm{logit}({\overset{\wedge }{Q^{*}}}^{1}(A,W))=\mathrm{logit}({\overset{\wedge }{Q^{*}}}^{0}(A,W))+\overset{\wedge }{ɛ}\overset{\wedge }{h}(A,W)$$

Here, $\overset{\wedge }{ɛ}$ can be estimated by logistic regression, where the mean of the outcome, bounded between 0 and 1, is modelled with a quasi-binomial distribution, by regressing $Y^{*}$ on $\overset{\wedge }{h}(A,W)$ with offset $\mathrm{logit}({\overset{\wedge }{Q^{*}}}^{0}(A,W)).$ ${\overset{\wedge }{Q}}^{1}(A,W)$ can be obtained by back-transforming ${\overset{\wedge }{Q^{*}}}^{1}(A,W)$ to the original scale. The resulting targeted estimates of the potential outcomes ${\overset{\wedge }{Q}}^{1}(0,W)$ and ${\overset{\wedge }{Q}}^{1}(1,W)$ are applied in the G-computation formula in order to obtain the TMLE

$${\overset{\wedge }{\psi }}^{\mathrm{TMLE}}=\frac{1}{N}\sum_{i=1}^{N}{\overset{\wedge }{Q}}^{1}(1,W_{i})-{\overset{\wedge }{Q}}^{1}(0,W_{i})$$

TMLE can use predictions from any fixed parametric model for the initial Q(·) (e.g. OLS or GLM) and g(·) (e.g. logistic regression). However, with machine learning methods, TMLE has been shown to reduce bias when the models for the assignment mechanism and the endpoint are misspecified.^16^ As in the previous sections, we consider super learning for the initial Q(·) and boosted CARTs for g(·).

#### 2.3.2 BCM

It is generally recommended that matching methods are followed by regression adjustment.^19,22,44^ The idea is similar to regression adjustment in randomised trials: regression is used to ‘clean up’ residual imbalances between treatment groups after matching.^51^ BCM^20,62^ adjusts the imputed potential outcome with the difference in the predicted endpoint that can be attributed to covariate imbalance between the matched pairs. These predictions are obtained using a regression of the endpoint on covariates, stratified by treatment assignment. The bias-corrected predictions of the potential outcomes are obtained as follows

$$\hat{Y}(0,W_{i})=\{Y_{i}\text{if}A_{i}=0\frac{1}{M}\sum_{j\varepsilon \zeta_{M}(i)}Y_{j}+\hat{Q}(0,W_{i})-\hat{Q}(0,W_{j})\text{if}A_{i}=1$$

$$\hat{Y}(1,W_{i})=\{\frac{1}{M}\sum_{j\varepsilon \zeta_{M}(i)}Y_{j}+\hat{Q}(1,W_{i})-\hat{Q}(1,W_{j})\text{if}\;A_{i}=0Y_{i}\text{if}\;A_{i}=1$$

For example, for an individual *i* who receives control, the imputed potential outcome under the treatment state is the average observed outcome of the *M* closest matches from the treatment group (indexed by *j*), adjusted with the difference between the predicted outcomes when covariate values are set to those of its own values, $\overset{\wedge }{Q}(1,W_{i})$ and the covariate values of the match, $\overset{\wedge }{Q}(1,W_{j})$. The corresponding estimator is the mean difference of these bias-corrected predicted potential outcomes

$${\overset{\wedge }{\psi }}^{\mathrm{BCM}}=\frac{1}{N}\sum_{i=1}^{N}\overset{\wedge }{Y}(1,W_{i})-\overset{\wedge }{Y}(0,W_{i})$$

BCM is consistent if Q(0,W) and Q(1,W) are consistently estimated^20^ or when the PS is correctly specified. Matching can decrease the sensitivity of estimates to the misspecification of the endpoint regression model^5^ and, for moderately non-linear response surfaces, adjustment even with a misspecified OLS model can reduce bias.^19–22^ Because an OLS regression, even including non-linear terms, might not capture highly non-linear response surfaces, we consider super learning for predicting the potential outcomes, as well as fixed parametric models. We implement 1-to-1 matching because increasing the number of matches would result in larger distances between matched treated and control units, and therefore increase bias.^29,51,63^ We match on the linear predictor of PS with replacement, allowing for ties. We estimate the PS using logistic regression and also using boosted CARTs.

## 3 Motivating case study

### 3.1 Overview

We consider the methods in a case study that evaluates the effect of alternative hip prosthesis types on the HRQoL of patients with osteoarthritis using an observational database on patients with THR. THR is one of the most common surgical procedures in the UK, with over 50,000 hip procedures performed in the National Health Service (NHS) in England and Wales in 2011;^64^ health care decision makers have a considerable interest in evaluating the effectiveness of different prosthesis types in routine care.^64^ A large-scale survey that collects PROMs on all patients who undergo elective surgery in the NHS provides a key data source for this evaluation. The resulting observational dataset includes pre- and post-operative HRQoL for patients with THR procedures.^30,31^

The dataset measures the HRQoL endpoint as EQ-5D-3L scores.^65^ The EQ-5D-3L is a generic instrument with five dimensions of health (mobility, self-care, usual activities, pain and discomfort, anxiety and depression) and three levels (no problems, some problems, severe problems). The EQ-5D-3L profiles were combined with health state preference values from the UK general population to give utility index scores on a scale ranging from 1 (perfect health), through 0 (death) to the worst possible health state, −0.59.^56^ This results in a bounded, semi-continuous distribution of the endpoint that exhibits a point mass at 1, posing a challenge for the specification of the regression model.^6^

A previous analysis of this dataset^66^ reported the relative effectiveness of common prosthesis types, such as cemented, cementless and ‘hybrid’ prostheses. The analysis used multivariate matching and linear regression to adjust for confounding and found a small but statistically significant advantage of hybrid compared to cementless prostheses.

For this motivating example, we estimate the ATE on EQ-5D-3L, 6 months after THR in males patients, aged 65–74 (*n* = 3583) who received hybrid versus cementless hip prostheses. We illustrate the use of TMLE and BCM with fixed parametric models and then machine learning estimation techniques, and compared to regression, matching, IPTW and WLS.

### 3.2 Statistical methods in the case study

Potential confounders include patient characteristics such as age, sex, body mass index, pre-operative health status (‘Oxford Hip score’ and HRQoL), comorbidities, disability, index of multiple deprivation and characteristics related to the intervention, such as surgeon experience (senior surgeon or not) and hospital type (NHS, private sector hospital or treatment centre). Of the 3583 patients included in the analysis, 70% had a missing value on at least one variable, with 46% having missing values for more than one covariate. Thirty-two per cent were missing data on post-operative HRQoL and 39% on BMI. Other covariates were complete for over 90% of the sample. Multiple imputation using chained equations was applied to impute missing covariate and endpoint values.^66^ Following recommendations,^67^ five multiply imputed datasets were created, and the analysis described below was performed on each dataset. Point estimates and variances were combined using Rubin’s formulae.^68^ Fixed parametric approaches for estimating Q(·) included OLS regression and a two-part model which can account for the point mass in the observed distribution of the endpoint.^6,69,70^ Here the binary part P(Y<1) was modelled with logistic regression, while a gamma regression was used for modelling the continuous part (Y'=1-Y when Y<1). Continuous covariate effects were modelled flexibly using smooth functions which are approximated by a linear combination of known spline basis functions and regression parameters. Such parameters were estimated by fitting generalised additive models using the R package ‘splines’, with default degrees of freedom of 4.^71^

For machine learning estimation of Q(·), we used the R package ‘Super Learner’,^72^ where the user-defined library included the following prediction algorithms: ‘glm’ (main terms linear regression), ‘glm.interaction’ (glm with covariate interactions) and a package that implements multivariate adaptive polynomial spline regression methods, ‘polymars’.^73^ Machine learning estimation of g(·) relied on boosted (logistic) CARTs, using the R package ‘twang’,^74^ with tuning parameters recommended by the developers.^24,50^ This implementation aimed to minimise mean covariate imbalance measured using Kolmogorov–Smirnov tests, reweighted by the estimated IPT weights.

We applied WLS with IPT weights obtained from the logistic model and also from the boosted CARTs. TMLE used the known minimum and maximum values of the endpoint as bounds, −0.59 and 1.^56^ Standard errors and 95% CIs were calculated using the sandwich estimator for IPTW and WLS, and using the influence curve^14^ for TMLE. For the matching methods, estimated standard errors took into account the matching process, but were conditional on the estimated PS, hence did not account for the uncertainty in the process of estimating the PS.^20,44^ For the two-part model and the super learning regression estimator, standard errors were estimated with the non-parametric bootstrap.^75^

### 3.3 Case study results

Table 1 presents balance on the main pre-operative characteristics of patients who underwent hybrid versus cementless THR, reported as absolute standardised mean differences. Patients with hybrid hip replacement were slightly older, had more comorbidities and were less likely to have been treated by a consultant or in a treatment centre.

**Table 1.** Balance on pre-operative characteristics, means and % standardised mean differences.

Covariate	Mean hybrid (*n* = 631)	Mean cementless (*n* = 2952)	SMD (%)
Age	69.7	69.3	15.98
Oxford hip score^a^	20.2	19.9	2.83
Pre-operative EQ-5D^a^	0.401	0.399	0.63
Index of deprivation^a^	3.26	3.03	15.92
ASA grade 1 (%)^a^	0.0903	0.120	9.55
ASA grade 2 (%)	0.740	0.738	0.52
Disability score	0.617	0.596	4.19
Obese^a^	0.270	0.266	0.69
Morbidly obese^a^	0.104	0.111	4.30
Number of comorbidities	1.00	0.96	4.14
Comorbidities
Heart disease	0.176	0.15	7.86
High bp	0.399	0.422	4.55
Stroke	0.0285	0.0169	7.78
Circulation	0.0777	0.0671	4.08
Lung disease	0.0555	0.0640	3.61
Diabetes	0.130	0.123	2.20
Kidney disease	0.0127	0.0207	6.24
Nervous system	0.00634	0.0118	5.20
Liver disease	0.0951	0.00339	7.65
Cancer	0.0602	0.0515	3.80
Depression	0.0491	0.0373	5.84
Consultant	0.803	0.869	17.64
Treatment centre	0.0491	0.122	26.16

Note: SMD: standardised mean difference. SMD was calculated as $d=100*\frac{\left|{\bar{x}}_{h}-{\bar{x}}_{c}\right|}{\sqrt{\frac{s_{h}^{2}+s_{c}^{2}}{2}}}$, where ${\bar{\text{x}}}_{\text{h}}$ and ${\bar{\text{x}}}_{\text{c}}$ are the means for the hybrid and cementless group, while the denominator includes the pooled standard deviation of the two groups, for a given covariate. Variables are dichotomous, with the exception of age, Oxford hip score, pre-operative EQ-5D-3L score, index of deprivation and number of comorbidities.

a Variables with missing values. Here, SMDs were combined using Rubin’s formulae.

**Figure 1.**
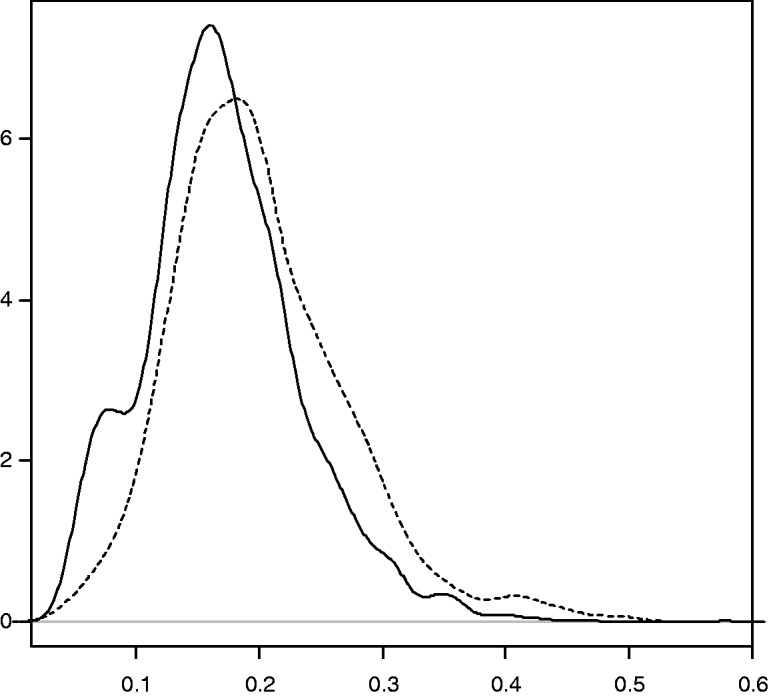
Densities of the estimated PS using logistic regression, hybrid versus cementless THR. Hybrid (dashed line) versus cementless (black line).

There was good overlap between the densities of the estimated PSs for the hybrid and cementless groups, when g(·) was obtained by logistic regression (Figure 1). The plots obtained using boosted CART for estimating the PS were similar.

**Figure 2.**
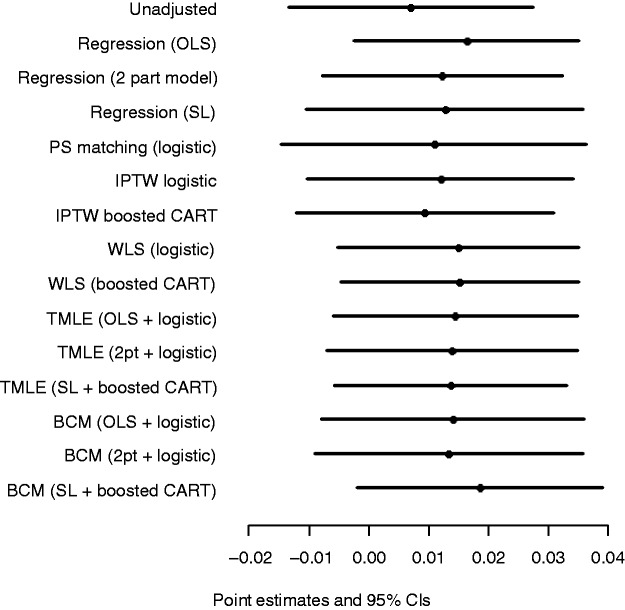
Point estimates and 95% CIs of ATE in terms of EQ-5D-3L score, hybrid versus cementless THR, across statistical methods. SL: super learner.

Figure 2 shows the point estimates and 95% CIs after combining the estimates obtained for the imputed datasets. All methods reported a small positive advantage in mean EQ-5D-3L scores for hybrid versus cementless prostheses, but with CIs that included zero.

## 4 Simulation study

### 4.1 Overview

The simulation study compares the performance of BCM and TMLE, in estimating the ATE of a binary treatment on an endpoint with a non-linear response surface. As in the case study, we compared these methods to regression, PS matching, IPTW and WLS, and for each method, we considered fixed parametric models and machine learning estimation for Q(·) and g(·). Motivated by the case study and previous simulation studies,^6,16,26^ we considered DGPs with non-linear response surfaces; normal, gamma-distributed and semi-continuous endpoints; good and poor overlap and with moderate and strong association between confounders and the endpoints. These DGPs (Table 2) were selected to highlight the differences between the performances of the methods under realistic circumstances, by investigating the following hypotheses:

1. Reweighting methods are anticipated to outperform BCM when overlap is good.^21^ In such scenarios, TMLE is expected to outperform BCM in terms of bias and efficiency.
2. When overlap is poor, BCM is expected to outperform TMLE, because matching can be less sensitive than weighting to extreme PS values and to the misspecification of g(·).^21,24,43^
3. Using appropriate machine learning methods is anticipated to reduce bias compared to using misspecified parametric models for Q(·) and g(·),^16,26^ across all methods considered.

We assumed a PS mechanism that generated good overlap of the densities of the true PS (DGP 1 and 2) and one that generated poor overlap (DGP 3–5). We considered moderate (DGP 1) and strong (DGP 2–5) association between the confounders and the endpoints. DGPs 1–3 considered a normal endpoint with an identity link function between the linear predictor and the endpoint, DGP 4 considered an endpoint which followed a gamma distribution with a log link, while DGP 5 considered a semi-continuous distribution, with a mixture of a beta-distributed random variable and values of 1.

**Table 2.** Summary of DGPs used in the simulation study.

Overlap	Confounder–endpoint association	Endpoint distribution
DGP 1	Good	Moderate	Normal
DGP 2	Good	Strong	Normal
DGP 3	Poor	Strong	Normal
DGP 4	Poor	Strong	Gamma
DGP 5	Poor	Strong	Semi-continuous

For each DGP, five scenarios were considered: (a) when fixed parametric models were used for both the PS and the endpoint regression, and these were correctly specified, (b and c) when one of the two was misspecified and (d) when the correct specification for both models was unknown. Scenario (d) had two sub-scenarios, differentiated by the implementation of the methods: in scenario (d1), we considered misspecified, fixed parametric models, while for scenario (d2) we considered machine learning estimates of Q(·) and g(·). Here, similarly to (d1), the correct parametric models were unknown for the investigator and were not included among the candidate prediction algorithms. For DGP 1, we report results from each of the five scenarios, while for DGPs 2–5, we only report the results for scenarios (d1) and (d2), as these were a priori judged the most realistic. The results for the remaining scenarios are available upon request.

Bias, variance, RMSE and the coverage rate for nominal 95% CIs of the estimated ATEs were obtained. Relative bias was calculated as the percentage of the absolute bias of the true parameter value, where absolute bias is the difference between the true parameter value and the mean of the estimated parameter. The RMSE was taken as the square root of the mean squared differences between the true and estimated parameter values.

### 4.2 DGPs

For each DGP, we generated 1000 datasets of *n* = 1000, with binary (*W*_1_ to *W*_5_) and standard normally distributed covariates $(W_{6}$ to $W_{8})$. This mix of binary and continuous covariates reflects the case study. The correlation coefficients between the covariates were set between 0.075 and 0.6. All covariates were true confounders, i.e. they influenced both the treatment assignment and the endpoint. Treatment was assigned according to a true PS that, like previous simulation studies, included main terms, higher order terms and interactions.^26,43^

**Figure 3.**
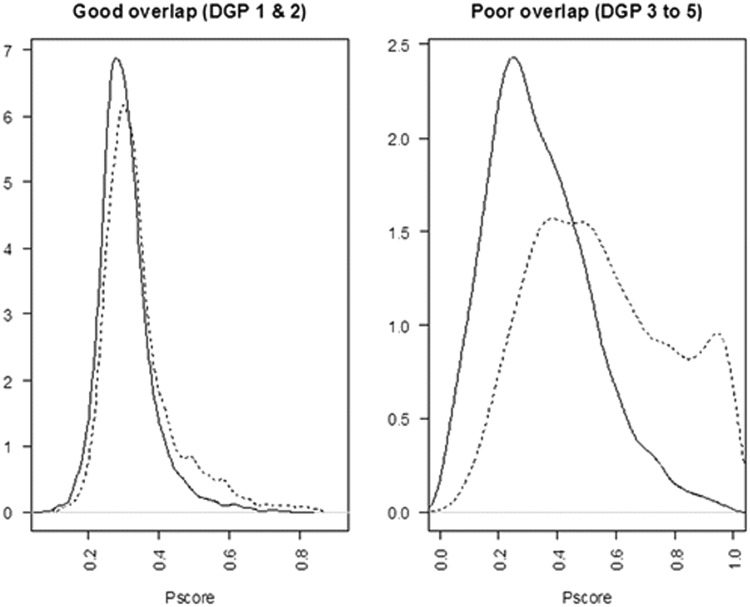
Densities of the true PS in the simulations for a typical sample (*n* = 10,000). Treated (dashed line) versus control (black line). (a) Good overlap (DGP 1 and 2), (b) poor overlap (DGP 3–5).

For DGP 1, the PS model resulted in a good overlap of the true PS (see Figure 3)

$$\mathrm{logit}(\mathrm{PS})=-1+k_{1}(0.3W_{1}-0.1W_{2}-0.2W_{3}+0.4W_{4}+0.7W_{5}\;+0.2W_{6}+0.2W_{7}-0.25W_{8}+0.8W_{6}^{2}-0.3W_{7}^{2}\;-0.3W_{8}^{2}-0.05W_{1}W_{2}-0.05W_{1}W_{3})$$

where $k_{1}=0.3$.

The treatment variable *A* was drawn from a Bernoulli distribution, using the PS as the parameter of success probability. The endpoint was drawn from a normal distribution with mean

$$\mu_{\mathrm{norm}}=15+0.4A+k_{2}(1W_{1}-0.1W_{2}+0.1W_{3}-0.1W_{4}+0.1W_{5}\;-0.1W_{6}+0.1W_{7}+0.1W_{8}-0.2W_{6}^{2}-0.1W_{7}^{2}-0.1W_{8}^{2}\;+0.2W_{6}^{3}+0.1W_{7}^{3}+0.1W_{8}^{3}-0.1W_{1}W_{2}+0.5W_{1}W_{7})$$

standard deviation of 1 and $k_{2}=1$.

In DGP 2, setting $k_{2}$ to 4 increased the strength of the confounder–endpoint association. In DGP 3, changing $k_{1}$ to 1 created a poor overlap of the true PS distributions (see Figure 3).

In DGP 4, the endpoint was drawn from a gamma distribution, with a log link, shape parameter of 100 and a scale parameter of $\frac{\mu_{\text{gam}}}{100}$, where the linear predictor was

$$\log(\mu_{\text{gam}})=3+0.2A-0.2W_{1}+0.2W_{2}-0.2W_{3}+0.5W_{4}-1W_{5}+0.5W_{6}-0.5W_{7}+0.2W_{8}\;-0.2W_{6}^{2}-0.01W_{7}^{2}-0.01W_{8}^{2}-0.01W_{6}^{3}-0.01W_{7}^{3}-0.01W_{8}^{3}-0.01W_{1}W_{2}-0.4W_{6}W_{7}$$

In DGP 5, motivated by the case study and previous simulation studies,^6^ the endpoint was generated as a mixture of a beta distributed continuous variable $Y^{'}$ and 1, using a Bernoulli distribution with parameter *p* to select between values from the two distributions

Y~(1-p)*1+p(1-Y')

where

$$\mathrm{logit}(p)=4-1A-0.2W_{1}+0.5W_{2}-0.5W_{3}-1W_{4}-0.3W_{5}\;+0.2W_{6}+0.5W_{7}-0.5W_{8}$$

$$Y'\sim \mathrm{Beta}(\mu_{\mathrm{beta}}*\mathrm{phi},\mu_{\mathrm{beta}}*(1-\mathrm{phi})),$$

$$\mathrm{logit}(\mu_{\mathrm{beta}})=-1-0.2A-0.5W_{1}-0.5W_{2}-0.5W_{3}+0.5W_{4}-0.5W_{5}-0.5W_{6}\;-0.5W_{7}-0.5W_{8}-0.2W_{6}^{2}-0.2W_{7}^{2}-0.2W_{8}^{2}-0.2W_{6}^{3}-0.2W_{7}^{3}-0.2W_{8}^{3}\;-0.2W_{1}W_{2}-0.2W_{6}W_{7})$$

The resulting semi-continuous distribution with a point mass at 1 reflects the observed endpoint in the case study. The true ATE was 0.4 in DGP 1–3, it was 9.98 for DGP 4 and 0.062 for DGP 5. While for DGPs 1–3 the treatment effect was constant across individuals, for DGP 4 and 5, the true ATE was obtained by simulating both potential outcomes for each individual, and taking the mean of the individual-level additive treatment effects.

### 4.3 Implementation of the methods

Correct specification was defined as applying a fixed parametric model according to the known features of the true DGP, such as the link function, the functional form between the covariates and the linear predictor, and the error distribution. For each DGP, the misspecified parametric g(·) and Q(·) models were logistic and OLS regressions with main terms only. Machine learning estimation of g(·) and Q(·) was as described in Section 3. The WLS estimator was implemented with main terms only, hence in this estimator the Q(·) component is misspecified. For the DGPs with poor overlap, in a sensitivity analysis we modified the IPTW, WLS and TMLE estimators, and used weights based on g(·) truncated at fixed levels of 0.025 and 0.975. For calculating coverage rates of nominal 95% CIs, standard errors were obtained as described in Section 3.

### 4.4 Simulation study results

**Figure 4.**
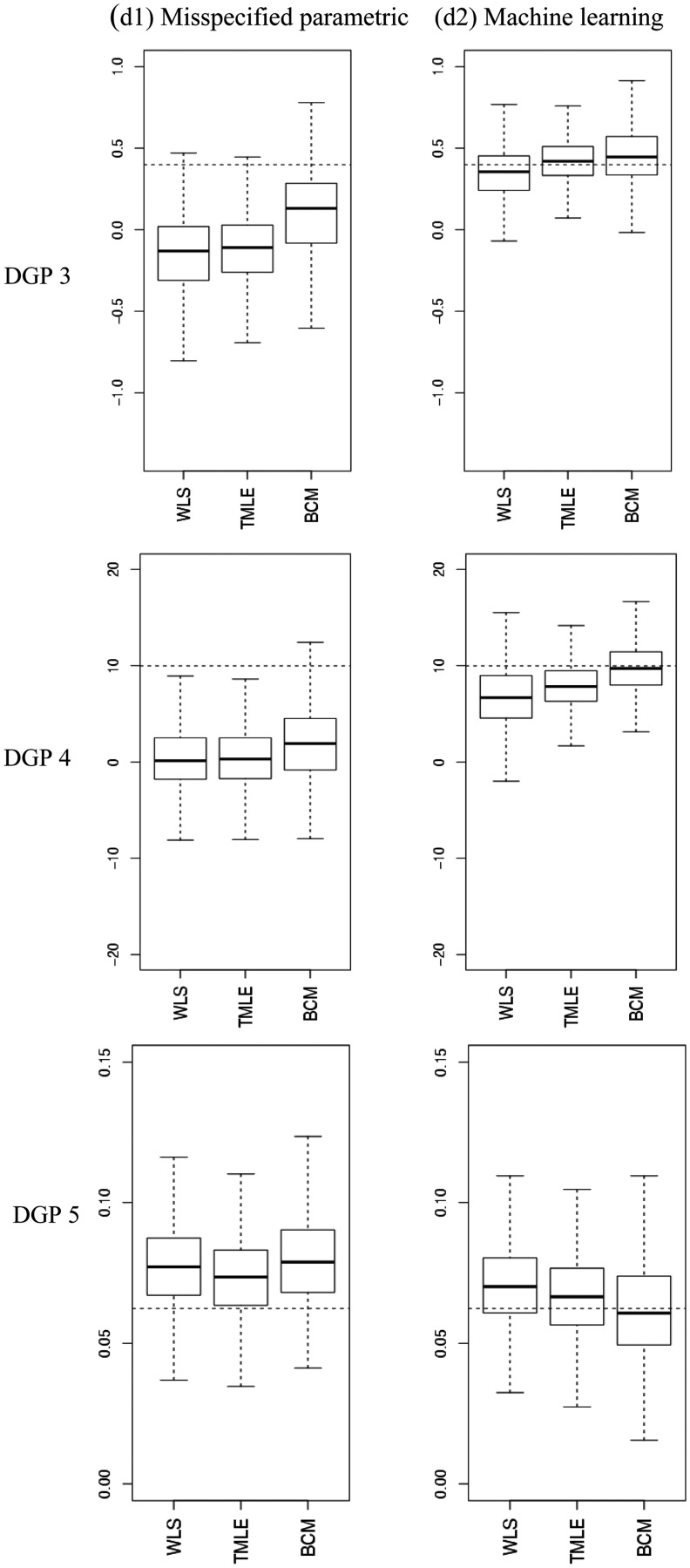
Estimated ATEs in the simulations. The boxplots show bias and variation, as median, quartiles and 1.5 times interquartile range for the estimated ATEs across 1000 replications. The dashed lines are the true values. The left panel provides results for when the PS model and endpoint were estimated with misspecified fixed parametric methods (d1), the right panel for when machine learning estimation (d2) was used. (a) DGP 3, (b) DGP 4, (c) DGP 5.

Tables 3 to 5 report the relative bias (%), variance, RMSE and 95% CI coverage for the estimators considered, and Figure 4 presents the distribution of the estimated ATEs with box plots.

**Table 3.** Simulation results for DGP 1, over 1000 replications: normal endpoint, moderate association confounder–endpoint association, good overlap.

Scenario	Relative bias (%)	Variance	RMSE	95% CI coverage (%)
*(a) Q correct – g correct*
OLS	−0.1	0.005	0.070	95
IPTW	0.5	0.008	0.091	99
PS matching	1.2	0.011	0.106	98
TMLE	−0.1	0.005	0.071	95
BCM	−0.1	0.007	0.082	95
*(b) Q correct – g misspecified*
OLS	−0.1	0.005	0.070	95
IPTW	−15.0	0.008	0.110	97
PS matching	−8.1	0.013	0.117	96
TMLE	−0.2	0.005	0.070	94
BCM	0.7	0.007	0.085	93
*(c) Q misspecified – g correct*
OLS	−11.7	0.008	0.098	90
IPTW	0.5	0.008	0.091	99
PS matching	1.2	0.011	0.106	98
WLS	0.6	0.008	0.087	95
TMLE	0.6	0.008	0.087	95
BCM	0.7	0.009	0.097	95
*(d1) Q and g misspecified parametric*
OLS	−11.7	0.008	0.098	90
IPTW	−15.0	0.008	0.110	97
PS matching	−8.1	0.013	0.117	96
WLS	−12.7	0.008	0.103	90
TMLE	−12.9	0.008	0.104	90
BCM	−7.4	0.011	0.108	93
*(d2) Q and g machine learning*
Regression (Q super learner)	−3.1	0.006	0.079	95
IPTW (g boosted CART)	10.2	0.007	0.091	98
WLS (Q OLS, g boosted CART)	0.5	0.006	0.076	97
TMLE (Q SL, g boosted CART)	1.1	0.006	0.074	94
BCM (Q SL, g boosted CART)	2.1	0.008	0.092	95

Note: In DGP 1 the true ATE was 0.4 and the bias using a naive estimator based on the mean difference was 20%. WLS is implemented as main terms only in regression; hence it is reported as a misspecified estimator.

**Table 5.** Simulation results for DGP 4 and 5, over 1000 replications: Normal and gamma endpoints, strong confounder–endpoint relationship, poor overlap.

Relative bias (%)	Variance	RMSE	95% CI coverage (%)
**DGP 4: Gamma endpoint, strong confounder–endpoint association, poor overlap**				
*(d1) Q and g misspecified parametric*				
OLS	−93.3	10.175	9.843	16
IPTW	−102.7	11.850	10.817	34
PS matching	−85.6	19.120	9.595	59
WLS	−96.9	11.475	10.252	19
TMLE	−96.4	10.303	10.140	17
BCM	−80.7	17.642	9.085	37
*(d2) Q and g machine learning*
Regression (Q super learner)	−11.8	7.600	2.998	90
IPTW (g boosted CART)	−80.1	16.585	8.974	62
WLS (Q OLS, g boosted CART)	−32.1	11.024	4.612	81
TMLE (Q SL, g boosted CART)	−20.7	6.115	3.224	70
BCM (Q SL, g boosted CART)	−2.5	6.755	2.610	98
**DGP 5: Semi-continuous endpoint, strong confounder–endpoint association, poor overlap**				
*(d1) Q and g misspecified parametric*				
OLS	26.0	0.0002	0.022	78
IPTW	15.0	0.0003	0.019	99
PS matching	26.9	0.0004	0.026	93
WLS	23.9	0.0003	0.022	83
TMLE	17.9	0.0002	0.019	90
BCM	27.1	0.0003	0.024	82
*(d2) Q and g machine learning*
Regression (Q super learner)	13.5	0.0002	0.017	91
IPTW (g boosted CART)	59.4	0.0003	0.041	72
WLS (Q OLS, g boosted CART)	12.9	0.0002	0.017	90
TMLE (Q SL, g boosted CART)	7.2	0.0002	0.016	87
BCM (Q SL, g boosted CART)	−1.1	0.0004	0.019	95

Note: In DGPs 4 and 5, the true ATE was 9.98 and 0.062, respectively. The bias using a naive estimator based on the mean difference was 170 and 150%, respectively.

Table 3 reports results for DGP 1, when there was good overlap, with a moderate association between the confounders and a normally distributed endpoint. When both Q(·) and g(·) were correctly specified, all methods reported minimal bias, with parametric regression (OLS with non-linear terms) and TMLE reporting the lowest RMSE. Regression, TMLE and BCM all provided coverage at the nominal 95%, while IPTW and PS matching reported coverage rates higher (98 and 99%) than the nominal level. When only one of the PS or endpoint model was misspecified, BCM and both DR methods (WLS and TML) remained unbiased. With dual misspecification, each method reported moderate levels of bias, but when machine learning estimation was used for Q(·) and g(·), bias was reduced to close to zero for all the methods that combined these components, with WLS and TMLE providing estimates with the lowest RMSE.

In DGP 2, with misspecified fixed parametric methods, stronger association between the confounders and the endpoint led to higher biases, but with machine learning estimation the bias for the methods that combined g(·) and Q(·) was again below 10% (Table 4). WLS and TMLE reported lower bias and RMSE than BCM. In DGPs 3–5, where there was poor overlap, with misspecified fixed parametric models, each method reported high bias. For each of these DGPs, machine learning estimation improved performance of the methods that combined g(·) and Q(·). In DGP 3, TMLE provided the lowest bias and RMSE, albeit with CI coverage that was lower than the nominal level (Table 4). In DGP 4 where we considered an endpoint with a gamma distribution, with machine learning approaches BCM showed less relative bias (2.5%) and RMSE than TMLE (20.7%), however higher variance (Table 4). In DGP 5, where we considered an endpoint with a semi-continuous distribution, TMLE and BCM with machine learning estimation performed best; BCM gave the lowest relative bias (1.1% versus 7.2%) and best CI coverage whereas TMLE reported the lowest RMSE and variance (Table 5).

**Table 4.** Simulation results for DGP 2 and 3, over 1000 replications: normal endpoint, strong confounder–endpoint association, good and poor overlap.

Relative bias (%)	Variance	RMSE	95% CI coverage (%)
**DGP 2: Normally distributed endpoint, strong confounder–endpoint association, good overlap**				
*(d1) Q and g misspecified parametric*
OLS regression	−45.9	0.052	0.292	86
IPTW	−59.1	0.067	0.350	98
PS matching	−34.0	0.099	0.342	96
WLS	−50.2	0.059	0.315	87
TMLE	−45.7	0.041	0.272	86
BCM	−31.4	0.074	0.299	90
*(d2) Q and g machine learning*
Regression (Q super learner)	−8.6	0.025	0.162	96
IPTW (g boosted CART)	41.0	0.036	0.251	99
WLS (Q OLS, g boosted CART)	2.6	0.022	0.149	100
TMLE (Q SL, g boosted CART)	3.1	0.011	0.106	95
BCM (Q SL, g boosted CART)	9.8	0.029	0.174	98
**DGP 3: Normally distributed endpoint, strong confounder–endpoint association, poor overlap**				
*(d1) Q and g misspecified parametric*
OLS regression	−119.2	0.050	0.527	40
IPTW	−160.6	0.082	0.703	71
PS matching	−81.1	0.100	0.453	84
WLS	−137.9	0.063	0.606	39
TMLE	−129.7	0.046	0.561	35
BCM	−73.8	0.072	0.399	74
*(d2) Q and g machine learning*
Regression (Q super learner)	−22.0	0.046	0.233	94
IPTW (g boosted CART)	100.6	0.034	0.442	82
WLS (Q OLS, g boosted CART)	−12.8	0.025	0.165	99
TMLE (Q SL, g boosted CART)	5.6	0.019	0.139	87
BCM (Q SL, g boosted CART)	12.3	0.034	0.191	98

Note: In DGPs 2 and 3, the true ATE was 0.4 and the biases, using a naive estimator based on the mean difference, were 80 and 190%, respectively.

IPTW using machine learning weights often reported high bias: for example for DGP 5, it reported higher bias than using a misspecified, fixed logistic regression. This indicated that using boosted CARTs for estimating the PS was insufficient to eliminate bias. For DGPs 3–5, where overlap was poor, truncating the weights for IPTW and TMLE for either the logistic or the boosted PS models did not change the results.

## 5 Discussion

This paper finds that in circumstances when the parametric models for both the endpoint regression function and PS are misspecified, both TMLE and BCM can reduce bias when coupled with machine learning methods.

We considered these methods, alongside more traditional PS, regression and DR methods in a case study that evaluated the effect of alternative types of THR for patients with osteoarthritis. This study illustrates a general challenge which is to specify a regression model for a non-normal endpoint (HRQoL), with a non-linear response surface. In the simulation studies, grounded in the case study, we generated endpoints data with normal, skewed and semi-continuous distributions, with non-linear covariate–endpoint relationships. In the simulated scenarios, where there was dual misspecification, and machine learning techniques were used to estimate the endpoint regression function and the PS, both TMLE and BCM could greatly reduce bias, in contrast to the high bias where misspecified fixed parametric models were used.

We found that the relative advantage of TMLE versus BCM was dependent on the features of the DGPs considered. In favourable settings such as good overlap and moderate association between the confounder and the endpoint, TMLE outperformed BCM in terms of bias and precision. This result corresponds to previous work that found that reweighting estimators outperformed BCM under good overlap.^21^ In a more challenging setting, when overlap was poor, and there was a strong association between the confounders and the endpoint, we found a bias–variance trade-off between the methods under comparison: for non-normal endpoints, BCM showed less bias, but was more variable than TMLE. We followed recent recommendations when reporting CIs for matching estimators,^44^ and like previous studies, we found that they reported somewhat higher than nominal coverage.^20^

Our work extends the previous literature in several aspects. First, this is the first paper that compares the relative performance of BCM and TMLE, and also compares these methods to traditional approaches. Second, while BCM has been proposed with flexible approaches for estimating the endpoint regression function, previous studies used OLS for adjustment.^20,21^ This study considers super learning, a machine learning method for bias correction, and finds that when matching is based on a PS that was also estimated using machine learning (boosted CARTs), the bias due to model misspecification was greatly reduced. We find this result across a range of DGPs including highly non-linear response surfaces. Third, unlike previous studies that used machine learning only for selected combined methods such as TMLE,^16^ this paper took a systematic approach and evaluated the impact of using machine learning estimation for single methods, such as regression and IPTW, and for combined methods, such as TMLE and BCM.

Similarly to Kang and Schafer (2007),^12^ we find that combining the PS and endpoint regression from misspecified fixed parametric models does not reduce bias compared to using these models in single methods such as IPTW. In the scenarios considered in this study, it was the combined use of machine learning approaches for estimating the endpoint regression and the PS that helped eliminate most of the bias due to observed confounding.

This work has some caveats. The methods considered and the simulation settings all assume ‘no unobserved confounding’. Machine learning methods can augment but not necessarily replace subject-matter knowledge when selecting the set of confounders that need to be controlled for.^76^ In the case study, while we used a rich set of measured cofounders suggested by previous literature and clinical expert opinion,^66^ some unobserved confounders such as unobserved patient preferences for prosthesis types may prevail. The scope of this paper did not extend to comparing alternative machine learning approaches. We found that boosted CARTs for estimating the PS, a method that has been found to outperform logistic regression and alternative machine learning approaches,^24^ did not consistently reduce bias compared to misspecified logistic regression. Further machine learning approaches may be considered for the PS, such as random forests^24^ or neural networks.^8^ These approaches also have promising application for estimating the endpoint regression function.^26^ Any machine learning method relies on subjective choices of the user. For boosted CARTs, tuning parameters such as the shrinkage parameter needs to be selected.^50^

For estimating the outcome regression, we demonstrated the use of the super learner.^38^ A distinguishing feature of this ensemble prediction approach compared to other model selection approaches is that it combines many estimators, by selecting a combination of predictions from alternative prediction algorithms. That is, the super learner aims to provide a better fit to the data than relying on any one prediction algorithm. In the simulation study, in order to mimic a situation where the investigator does not know the true DGP, we required the super leaner to consider the same, restricted range of prediction algorithms (including GLMs, generalised additive models and multivariate adaptive polynomial spline regression) for each DGP. In practice, we recommend that the analyst requires the super learner to consider a richer set of prediction algorithms; a wide range of models and prediction algorithms should be proposed according to subject-matter knowledge to encourage the consistent estimation of the regression function albeit at the expense of increased computational time.^39^ These prediction algorithms can include advanced model selection methods such as fractional polynomials^77^ or penalised model selection approaches.^78^

This paper considered continuous and semi-continuous endpoints motivated by the case study. The approaches presented are in principle applicable to binary, count or survival outcomes and other parameters such as the odds ratio, risk ratio or hazard ratio. TMLE has been demonstrated to have good finite sample performance for binary and survival endpoints.^17,59^ While matching estimators have also been proposed for estimating risk ratios and odds ratios,^46,79^ BCM estimators for these parameters have not yet been developed.

In the simulations, each method is adjusted for the observed covariates known to be predictive of both treatment assignment and of the outcome. In practice, this feature of the DGP is not known, and subject-matter knowledge should be used to select for adjustment of those potential confounders that are measured before treatment, and are both predictive of treatment selection and the endpoint.^1^ The inclusion of those covariates which influence treatment assignment, but not the endpoint in the PS can lead to estimates that are statistically inefficient.^80–82^

This work also opens up areas for future research. In the common settings of poor overlap, an extension of TMLE, collaborative maximum likelihood estimation (C-TMLE)^55,83^ can outperform TMLE. C-TMLE uses machine learning to select a sufficient set of covariates for inclusion in g(·) that reduces bias while minimising overall mean squared error.

We conclude that both TMLE and BCM have the potential to reduce bias due to observed confounding, in common settings of dual misspecification, if coupled with machine learning methods for estimating the PS and the endpoint regression function. TMLE is implemented as a readily available software package.^61^ For BCM, the available packages currently allow for regression adjustment using OLS only.^62,84^ In order to facilitate the uptake of the methods, the Supplementary Appendix provides code for the implementation of TMLE and BCM with machine learning.

## Supplementary Material

Supplementary material
