# Supplementary material for: Evaluating treatment effectiveness under model misspecification: A comparison of targeted maximum likelihood estimation with bias-corrected matching
Source: Stat Methods Med Res. 2014 Feb 12;25(5):2315–36. doi: 10.1177/0962280214521341 (PMC5051604; doi:10.1177/0962280214521341)
Supplement: Supplementary material [file Supplementary_Material.pdf]

Web Appendix for the paper "Evaluating treatment effectiveness under model misspecification: a comparison of targeted maximum likelihood estimation with bias-corrected matching"

R code for the implementation of TMLE and BCM

This section provides code for the implementation of TMLE and BCM, coupled with machine learning estimation approaches proposed in the paper, using the R statistical software (1). We also present code for the machine learning methods: "super learning" for predicting the endpoint and boosted CARTs for estimating the PS. The user-written functions implemented here call some pre-written R routines, for example the `tmle` (2), `Matching` (3), `Super Learner` (4) and `twang` (5) packages. These packages need to be installed and loaded in the R workspace order to use the functions presented here.

First, the necessary libraries need to be loaded:

```
library(splines)
library(twang)
library(SuperLearner)
library(Matching)
```

Then, we define the data frame, `data`, used in the analysis. This dataset includes the endpoint `q2_eq5d_index`, the treatment indicator `Hyb` and the covariates.

```
data=as.data.frame(dataset_men_M1)
```

We create the design matrix that will be used by some of the corresponding user defined R functions:

```
designreg=glm( Hyb ~ age
         +  q1_score
         +  q1_eq5d_index
         +  factor(IMD)
```

```
                +  ASAgrade1
                +  ASAgrade2
                +  q1_disability
                +  obese
                +  morbobese
                +  heart_disease
                +  high_bp
                +  stroke
                +  circulation
                +  lung_disease
                +  diabetes
                +  kidney_disease
                +  nervous_system
                +  liver_disease
                +  cancer
                +  depression
                +  Consultant
                +  TC, family=binomial(link="logit"), data=data)

design=model.matrix(designreg)
```

We also define the formula for the PS:

```
boost.CART.form <- as.formula(Hyb ~ age
                +  q1_score
                +  q1_eq5d_index
                +  IMD
                +  ASAgrade1
                +  ASAgrade2
                +  q1_disability
                +  obese
                +  morbobese
                +  heart_disease
                +  high_bp
                +  stroke
                +  circulation
                +  lung_disease
                + diabetes
                +  kidney_disease
                +  nervous_system
                +  liver_disease
                +  cancer
                +  depression
                +   Consultant
                +   TC)
```

Estimating the PS

The function named `boost.CART.func` estimates a PS using boosted logistic CARTs. The function takes the arguments `formula`, which specifies the variables the user wants to include in the PS model, and `data`. The function sets the tuning parameters to the values

recommended by the developers (6), and maximises balance based on the mean of the KS statistic. The function returns the estimated PS, the linear predictor of the estimated PS, and the IPT weight.

```
boost.CART.func = function(formula, data) {

            boost.CART.ps=ps(formula=formula,
            data=data,
            shrinkage = 0.0005,
            n.trees=10000,
            interaction.depth=2,
            iterlim=20000,
            stop.method="ks.mean")

            w.boost.CART = boost.CART.ps$w
            ps.boost.CART = boost.CART.ps$ps
                  linpred.boost.CART=
predict.gbm(boost.CART.ps$gbm.obj,            data,
boost.CART.ps$n.trees)
            return(list(w.boost.CART=w.boost.CART,
ps.boost.CART=ps.boost.CART,  linpred.boost.CART=linpred.boost.CART))

}
```

By calling the function, we can obtain the estimated PS, and attach it to `data`:

```
res.boost<-boost.CART.func(boost.CART.form, data=data)

ps.boost = unlist(res.boost$ps.boost.CART)

data=cbind(data, ps.boost)
rm(ps.boost)
```

Creating PS matched data

Before BCM is performed, a matched dataset needs to be created. The function named `PSmatch.function` calls the `Match()` function (3), taking the following arguments: `data`, and `pscore`, the estimated propensity score. The function returns the original return object of the `Match()` function (3), which includes the matched dataset, here named `mtchout.Y`. The function also returns the matching frequency weights `K`, as well as transformed version of this vector, `Kprime`, later used for calculating the standard errors around for matching estimator (7).

```
PSmatch.function=function(data,pscore)  {

      mtchout.Y=Match(Y=data$q2_eq5d_index,Tr=data$Hyb,X=pscore,

            estimand="ATE", ties=TRUE)
            n <- length(data$q2_eq5d_index)
            K <- rep(0, n)
            names(K) <- 1:n
            Kprime <- K
            extra <- by(mtchout.Y$MatchLoopC[,3],
                   mtchout.Y$MatchLoopC[,2], sum)
            K[rownames(extra)] <-  extra
            Kprime.extra <- by(mtchout.Y$MatchLoopC[,3],
                        mtchout.Y$MatchLoopC[,2], function(x){sum(x^2)})
            Kprime[rownames(Kprime.extra)] <-  Kprime.extra
            return(list(mtchout.Y=mtchout.Y,K=K, Kprime=Kprime))
                                                                }
```

Now the function can be called to obtain the matched dataset, `ps.match.data.w.boost`, and the vectors of frequency weights (`K.boost` and `K.prime.boost`).

```
PS.match.object.boost=PSmatch.function(data,data$ps.boost)

ps.match.data.boost=PS.match.object.boost$mtchout.Y
ps.match.data.w.boost <-
rbind(data[ps.match.data.boost$index.treated,],
data[ps.match.data.boost$index.control,])

ps.match.data.w.boost <- cbind(ps.match.data.w.boost,
weights=c(ps.match.data.boost$weights,ps.match.data.boost$weights))

K.boost=PS.match.object.boost$K
K.prime.boost=PS.match.object.boost$Kprime
```

Predicting the expected potential outcome with the super learner

The user defined function named `my.SL.ate` predicts the expected potential outcomes under treated and control states, using the Super Learner (4). The function takes the arguments `W` (the covariates), `A` (the observed treatment), and `Y`, the observed endpoint. We use super learner for estimating two regressions functions, stratified by treatment, as suggested by Abadie and Imbens (2011). This provides the algorithm flexibility to select different models for estimating the potential outcomes under treatment and control states. The function returns the original "Super Learner" object that includes information on the final

models selected, `m0` for the regression function selected to estimate the potential outcome under control and `m1` under treatment. The predictions for the potential outcomes are stored in the matrix `Q.SL.object`, which includes two vectors, the predicted potential outcomes under control and treatment, each with the length of the number of individuals in the sample.

```
my.SL.ate=function(W,A,Y) {

      matrix <- data.frame(W)

       m0 <- SuperLearner(Y[A==0], matrix[A==0,], newX = matrix,
             SL.library =  my.SL.library.short,
             family = gaussian())

      m1 <- SuperLearner(Y[A==1], matrix[A==1,], newX = matrix,
              SL.library = my.SL.library.short,
             family = gaussian())

      Yhat.0 <- m0$SL.predict
      Yhat.1 <- m1$SL.predict

      Q.SL.object=cbind(Yhat.0,Yhat.1)
      ate_SL=mean(Yhat.1-Yhat.0)
      return(list(Q.SL.object=Q.SL.object,m0=m0,m1=m1))
                                                   }
```

This function can be extended to incorporate weights, the modified function is named `my.SL.ate.matchw`. This is necessary, because for the bias-corrected matching estimator it is recommended that regression predictions are obtained using data weighted with the matching frequency weights, `K` (7).

```
my.SL.ate.matchw=function(W,A,Y,K) {

 matrix <- data.frame(W)

 m0 <- SuperLearner(Y[A==0], matrix[A==0,], newX = matrix,
       SL.library =  my.SL.library.short,
       family = gaussian(),obsWeights=K[A==0])

 m1 <- SuperLearner(Y[A==1], matrix[A==1,], newX = matrix,
       SL.library = my.SL.library.short,
      family = gaussian(),obsWeights=K[A==1])

Yhat.0 <- m0$SL.predict
Yhat.1 <- m1$SL.predict

Q.SL.object=cbind(Yhat.0,Yhat.1)
ate_SL=mean(Yhat.1-Yhat.0)
```

```
      return(list(Q.SL.object=Q.SL.object,ate_SL=ate_SL,m0=m0,m1=m1))
}
```

Before calling the function, the super learner library, including all the prediction algorithms selected by the user, needs to be defined.

```
my.SL.library.short <-c("SL.glm","SL.glm.interaction", "SL.polymars")
```

Here we include the algorithms `"SL.glm", "SL.glm.interaction"` and `"SL.polymars".`

```
      SL.object=my.SL.ate(design,data$Hyb,data$q2_eq5d_index),
```

then with matching frequency weights.

```
      SL.object.BCM.boost   <-  my.SL.ate.matchw(design,data$Hyb,
                          data$q2_eq5d_index,K=K.boost)
```

Implementing BCM

The function `BCM.AI` implements the BCM estimator proposed by Abadie and Imbens (2011). The function takes the following objects: `Y` (the observed endpoint), `A` (the observed treatment), `d.match`, the matched data obtained from the PS matching, `Yhat.0,` the vector of predicted potential outcomes under control, and `Yhat.1`, the vector of predicted potential outcome under treatment, as well as the `K` and `Kprime` vectors, describing the matching frequency weights. The function returns the point estimate of the ATE, `tau,` and the estimated variance of the ATE`, AIvar.`

```
BCM.AI <- function(Y,A, d.match,Yhat.0,Yhat.1, K, Kprime) {
      Y_j.0 <- Y_j.1 <- Y
      Ycounterfactual <- by(Y[d.match$MatchLoopC[,2]],
                       d.match$MatchLoopC[,1], mean)
      Y_j.1[A==0] <- Ycounterfactual[A==0]
```

```
    Y_j.0[A==1] <- Ycounterfactual[A==1]

    mu_0.Xi <- Yhat.0
    mu_0.Xj <- by(Yhat.0[d.match$MatchLoopC[,2]],
               d.match$MatchLoopC[,1], mean)
    mu_1.Xi <- Yhat.1
    mu_1.Xj <- by(Yhat.1[d.match$MatchLoopC[,2]],
               d.match$MatchLoopC[,1], mean)

    Ytilde.0 <- Y_j.0
    Ytilde.1 <- Y_j.1
    Ytilde.0[A==1] <- Y_j.0[A==1] + mu_0.Xi[A==1] - mu_0.Xj[A==1]
    Ytilde.1[A==0] <- Y_j.1[A==0]  + mu_1.Xi[A==0] - mu_1.Xj[A==0]

    tau.bcm <- mean(Ytilde.1 - Ytilde.0)
    n <- length(Y)
    sigmasq.X <- 1/(2*n) * sum((Ytilde.1      - Ytilde.0 - tau.bcm)^2)

    var.SATE <- 1/n^2 *  sum(( 1 + K)^2 * sigmasq.X)
    var.PATE <- 1/n^2 * sum ( (Ytilde.1 - Ytilde.0 - tau.bcm)^2 +
               ( K^2 + 2*K - Kprime)*sigmasq.X)

    return(list(tau = tau.bcm, AIvar=max(var.SATE, var.PATE),
                                     var.PATE = var.PATE))
                                                                    }
```

The point estimate and CI around the ATE is estimated by calling the function:

```
BCM.SL.boost <- BCM.AI(dataset_men_M1$q2_eq5d_index,
                      dataset_men_M1$Hyb,
                      ps.match.data.boost,
                      SL.object.BCM.boost$Q.SL.object[,1],
                      SL.object.BCM.boost$Q.SL.object[,2],
                      PS.match.object.boost$K, PS.match.object.boost$Kprime)
```

The estimated ATE, with its standard error can be obtained as follows:

```
coef.BCM.SL.boost <- BCM.SL.boost$tau
se.BCM.SL.boost <- sqrt(BCM.SL.boost$AIvar)
```

Implementing TMLE

The R package `tmle()` offers an accessible implementation of TMLE (2). The `tmle()` function takes the arguments `Y` (the observed endpoint), `A` (the observed treatment), `W` (the design matrix), and `Q` (the two vectors of potential outcomes). As a default, the `tmle()` function applies logistic fluctuation, and bounds the endpoint between the observed minimum and maximum values (here, between -0.59 and 1).

```
tmle.SL.boost=tmle(Y=data$q2_eq5d_index,A=data$Hyb,W=design, Q=Q.SL,
g1W=data$pscore.boost)
```

The estimated ATE and its confidence intervals can be then obtained:

```
coef.tmle.SL.boost <- tmle.SL.boost$estimates$ATE$psi
ciU.tmle.SL.boost <- summary(tmle.SL.boost)$estimates$ATE$CI[2]
ciL.tmle.SL.boost <- summary(tmle.SL.boost)$estimates$ATE$CI[1]
```

References

1. R Development Core Team. R: A language and environment for statistical computing. Vienna, Austria: R Foundation for Statistical Computing; 2011.
2. Gruber S, van der Laan MJ. tmle: An R Package for Targeted Maximum Likelihood Estimation. Journal of Statistical Software. 2012;51(13):1-35.
3. Sekhon JS. Matching: multivariate and propensity score matching with automated balance search. Journal of Statistical Software. 2011(In Press).
4. Polley EC, van der Laan MJ. R package "SuperLearner". 2010; Available from: http://cran.r-project.org/web/packages/SuperLearner/index.html.
5. Ridgeway G, McCaffrey D, Morral A, Griffin BA, Burgette L. twang: Toolkit for Weighting and Analysis of Nonequivalent Groups. 1.2-5 ed2006.
6. McCaffrey D, Ridgeway G, Morral A. Propensity Score Estimation with Boosted Regression for Evaluating Causal Effects in Observational Studies. Psychol Methods. 2004;9(4):403-25.
7. Abadie A, Imbens GW. Bias-Corrected Matching Estimators for Average Treatment Effects. J Bus Econ Stat. 2011;29(1):1-11.
